# The prevalence of potentially traumatic events in childhood and associations with mental disorders, suicide and physical health in adulthood: An Australian nationally representative cross-sectional study

**DOI:** 10.1177/00048674251381004

**Published:** 2025-11-17

**Authors:** Emma L Barrett, Lucinda Grummitt, Sam Jones, Kirsty Rowlinson, Fotini Vasilopoulos, Maree Teesson, Katherine L Mills, Matthew Sunderland

**Affiliations:** The Matilda Centre for Research in Mental Health and Substance Use, The University of Sydney, Sydney, NSW, Australia

**Keywords:** Child trauma, mental health, suicide, physical health

## Abstract

**Objective:**

This study aimed to estimate the population prevalence of exposure to potentially traumatic events (e.g. serious accidents, physical or sexual violence and natural disasters) during childhood among Australians and examine associations between childhood potentially traumatic events and mental disorders, suicide and long-term physical health conditions.

**Methods:**

Survey data from the 2020 to 2022 National Survey of Mental Health and Wellbeing were analysed, which included a nationally representative household sample of Australians aged 16–85 years (*n* = 15,893).

**Results:**

Up to 42% of Australians (approx. 8,250,948) have been exposed to a potentially traumatic event prior to the age of 18 years. The more common types of potentially traumatic events experienced prior to 18 years were unexpected death of a loved one (27.5%), witnessing domestic violence (21.1%), sexual assault (21.0%) and witnessing serious injury or death (20.0%). Australians exposed to childhood potentially traumatic events had significantly higher odds of any lifetime *Diagnostic and Statistical Manual of Mental Disorders*, Fourth Edition mental disorders compared to those who had not experienced potentially traumatic events (adjusted odds ratio: 1.51, 95% confidence interval: 1.32–1.74). They also had higher odds of suicidal thoughts (adjusted odds ratio: 1.57, 95% confidence interval: 1.28–1.92), plans (adjusted odds ratio: 1.60, 95% confidence interval: 1.17–2.19) and attempts (adjusted odds ratio: 2.04, 95% confidence interval: 1.40–2.98) and higher odds of asthma, arthritis, cancer and kidney disease compared to those who had not experienced potentially traumatic events in their lifetime.

**Conclusion:**

Childhood potentially traumatic events are prevalent in the Australian general population and associated with serious mental and physical health conditions. These findings have important implications for early detection and intervention, trauma-informed healthcare approaches, and for policy and practice across health, education and social service systems.

## Introduction

Exposure to potentially traumatic events (PTEs) during childhood is common and substantially impacts the lives of millions globally (Benjet et al., 2016). PTEs encompass a range of situations where individuals perceive their safety or the safety of others is threatened (e.g. serious accidents, physical or sexual violence and natural disasters) (Karstoft and Armour, 2023). Childhood PTEs are a significant predictor of poor health, contributing to the onset of mental and substance use disorders, self-harm and suicide attempts, and multiple physical health conditions (Chandrasekar et al., 2023; Grummitt et al., 2021; Huang et al., 2015; Wiss and Brewerton, 2020).

Epidemiological evidence highlights the particularly damaging effects of childhood PTEs compared with PTEs experienced in adulthood (after 16 or 18 years of age). Childhood interpersonal trauma is associated with almost six times higher odds of any mental disorder compared to interpersonal trauma experienced in adulthood (Zlotnick et al., 2008). The extensive development that occurs during childhood makes this period particularly vulnerable to the impact of trauma, increasing risk of long-term health complications (Andersen and Teicher, 2008; Colich et al., 2020).

The current prevalence of childhood PTEs in the Australian resident population (hereafter referred to as ‘Australians’) remains unclear. The landmark Australian Child Maltreatment Study (ACMS) recently provided the first nationally representative estimates of childhood maltreatment (physical, sexual, emotional abuse, neglect and domestic violence), finding 62% of Australians had experienced childhood maltreatment (Higgins et al., 2023). However, there are many PTEs not captured by these data, such as natural disasters; serious accidents, illness or injury; and exposure to war or combat, that are associated with the onset of poor mental and physical health (Lewis et al., 2019; McFarlane, 2010). While conceptually related, PTEs also differ from adverse childhood experiences (ACEs), a framework widely used globally. ACEs typically include childhood maltreatment and household dysfunction (e.g. parental substance use problems, parental incarceration), but do not encompass broader traumatic events like natural disasters or serious accidents. Thus, PTEs represent potentially harmful experiences that may occur in a variety of contexts. The last investigation of the prevalence of PTEs in the Australian resident population comes from the National Study of Mental Health and Wellbeing (NSMHW) conducted in 2007, revealing that 75% of the Australian adult population had been exposed to PTEs in their lifetime (Mills et al., 2011; Sunderland et al., 2016). The prevalence of *childhood* PTEs, however, was not reported. There is a significant lack of up-to-date knowledge on the prevalence and impact of childhood PTEs among Australians. Moreover, there is currently no report of the *projected* lifetime prevalence of mental disorders for children exposed to PTEs in Australia. This information is crucial for understanding the scope and long-term burden of childhood PTEs.

This study aims to (1) estimate the prevalence of childhood PTEs among Australians and (2) examine associations between PTEs (mutually exclusive groups of childhood exposure, adult-only exposure, no exposure) and lifetime prevalence and projected lifetime prevalence of mental disorders, suicidality and long-term physical health conditions.

## Methods

### Sample

The 2020–2022 NSMHW was a nationally representative cross-sectional survey conducted by the Australian Bureau of Statistics (ABS). It utilised a household sampling strategy and involved recruitment of two separate cohorts (due to the COVID-19 pandemic) between 5 December 2020 to 31 July 2021 and 4 December 2021 to 31 October 2022. The ABS created a combined sample using these two cohorts with appropriate weights (Slade et al., 2024).

The sample was recruited through a two-stage sampling strategy, which involved the random selection of households from all Australian states and territories, followed by random selection of an individual within this household aged between 16 and 85 years old. The sample is broadly representative of the Australian population aged 16–85 years. The final sample size was 15,893 participants (53.4% females and 46.6% males), representing a 52% response rate. A detailed description of the sampling design, methodology and sample characteristics is provided elsewhere (ABS, 2020-2022, Slade et al., 2024). Trained ABS personnel conducted a structured, face-to-face interview with participants.

### Measures

#### Demographic characteristics

Participants were asked their age (years) and gender (female, male, non-binary, use a different term).

#### PTEs

Participants were asked whether they had experienced 26 specific PTEs, plus two questions to assess any other extremely traumatic or life-threatening event not listed (see Table 1). Any PTE that a participant reported was followed up with a question regarding the participant’s age at first exposure to that event. From this information, participants were deemed to have experienced childhood PTEs if they had been exposed to any PTE between 0 and 17 years. Participants who reported that their first age of PTE exposure was from age 18 onwards were classified as having experienced PTEs during adulthood only.

**Table 1.** Weighted lifetime prevalence of exposure and mean age of first exposure to different PTEs prior to 18 years of age among Australians (*n* = 15,893).

PTE	Weighted prevalence % (SE)	Age of first exposure, mean years (SD)
Combat exposure	0.13 (0.04)	16.50 (0.90)
Ever been in an unarmed civilian in a place where there was a war/revolution/invasion/military coup	4.05 (0.41)	7.42 (5.16)
Lived as a civilian in a place where there was ongoing terror of civilians for political/ethnic/religious/other reasons	4.12 (0.37)	7.82 (5.46)
Ever a refugee	2.06 (0.22)	7.16 (4.92)
Ever kidnapped or held captive	0.98 (0.14)	9.61 (4.61)
Exposed to a toxic chemical or substance that could cause serious harm	1.99 (0.22)	12.68 (4.64)
Ever involved in a life-threatening automobile accident	6.93 (0.44)	12.26 (4.78)
Whether had any other life-threatening accident, including on the job	3.00 (0.31)	11.30 (4.78)
Whether involved in a major natural disaster (like a devastating bushfire, flood, cyclone or earthquake)	12.06 (0.55)	10.63 (4.17)
Man-made disaster, like a fire started by a cigarette or bomb explosion	1.76 (0.20)	10.69 (4.61)
Ever had life-threatening illness	6.28 (0.45)	7.72 (5.47)
Ever badly beaten as a child by parents/guardians	11.66 (0.51)	6.88 (3.50)
Whether ever badly beaten by a spouse or romantic partner	1.65 (0.26)	15.99 (1.92)
Ever badly beaten by anyone else	7.88 (0.45)	12.43 (3.78)
Ever mugged/held up/threatened with a weapon	8.22 (0.47)	13.96 (3.17)
Ever raped	9.14 (0.37)	11.06 (4.35)
Ever sexually assaulted (excluding rape)	20.98 (0.67)	11.25 (3.98)
Ever stalked by someone	5.17 (0.33)	14.27 (2.77)
Ever experienced the unexpected death of someone close	27.47 (0.77)	12.25 (4.03)
Ever someone close ever had an extremely traumatic experience	7.98 (0.47)	11.44 (5.07)
Ever witnessed any serious physical fights at home when a child	21.09 (0.71)	7.13 (3.31)
Ever witnessed someone being badly injured/killed/unexpectedly saw a dead body	20.04 (0.68)	12.00 (3.77)
Ever did something that accidently led to the serious injury or death or another	0.70 (0.14)	12.28 (3.83)
Ever on purpose seriously injured/tortured/killed another person	0.27 (0.08)	14.63 (2.22)
Ever saw atrocities or carnage such as mutilated bodies or mass killings	2.26 (0.27)	11.51 (4.03)
Ever experienced any other extremely traumatic or life-threatening event (specified)	1.97 (0.21)	11.03 (4.17)
Ever experienced a traumatic or life-threatening event (private event)	3.20 (0.25)	10.49 (4.30)
Any trauma prior to 18 years	41.61 (0.54)	9.55 (4.76)
Multiple trauma types (>1) prior to 18 years	46.25 (0.85)^a^	–

SE: standard error; SD: standard deviation.

This table does not include any PTEs that occurred after age 18.

a This percentage was calculated among those with a childhood PTE (*n* = 6597) and reflects the percentage of those with a childhood PTE who experienced multiple PTEs before age 18.

#### Mental disorders

The World Mental Health Survey Initiative version of the Composite International Diagnostic Interview version 3.0 (WMH-CIDI 3.0) was used to assess mental disorders. Lifetime diagnoses as per the *Diagnostic and Statistical Manual of Mental Disorders*, Fourth Edition (*DSM*-IV) ‘without hierarchy’ were derived in order to capture the true extent of comorbidity. This captured diagnoses of selected anxiety disorders (panic disorder, agoraphobia, social phobia, generalised anxiety disorder, obsessive compulsive disorder [OCD], post-traumatic stress disorder [PTSD]), affective disorders (depressive episode, dysthymia, bipolar disorder) and substance use disorders (alcohol use disorder and drug use disorder). The presence of alcohol use disorder was assessed for participants who had consumed at least 12 standard drinks in any 12-month period and three or more standard drinks on at least one occasion during this period. That is, only those participants meeting the above criteria were administered the relevant diagnostic questions to determine whether they also met criteria for a lifetime alcohol use disorder. Drug use disorder was assessed in relation to four substance classes: stimulants, sedatives, opioids and cannabis and only for participants who had used the substance more than five times in their life.

#### Suicidal thoughts, suicide plans, attempts and self-harm

Participants were asked whether they had ever seriously thought about taking their own life. Those who responded yes were asked whether they had ever made a plan to take their own life and whether they have attempted to take their own life. Participants were also asked whether they had ever intentionally harmed themselves without intending to take their own life.

#### Physical health conditions

Participants were asked whether they had been told by a doctor or nurse that they currently had a long-term physical health condition, defined as a condition which had lasted or was expected to last for 6 months or more. Those who endorsed having a long-term physical health condition were asked which of the following conditions they had been diagnosed with arthritis; osteoporosis; asthma; cancer (including remission); dementia; diabetes (excluding during pregnancy); heart disease (including angina or past heart attack); effects of a stroke; chronic kidney disease; mental health condition (including depression or anxiety); bronchitis or emphysema; any other current, long-term health condition(s).

### Data analysis

To ensure the sample was representative of the general Australian population, data were weighted to conform to independent population estimates by state, part of state, age and gender. In addition, balanced repeated replicate weights were used to account for the complex survey sampling design when estimating standard errors (SEs) and confidence intervals (CIs).

Descriptive statistics, including the interquartile range for childhood PTEs, prevalence estimates and their SEs, were calculated. A series of logistic regressions were conducted to identify associations between childhood PTEs (CT; <18 years) and all outcomes (i.e. mental disorders, suicidality and physical health conditions) compared to those who had experienced PTEs only during adulthood (>17 years) and those who had not experienced any PTEs (i.e. no PTEs). Multivariable logistic regression analyses were carried out to control for age, gender and number of trauma types experienced, and these factors were found to be significantly different across comparison groups (see Table 1). SEs, odds ratios (ORs) and 95% confidence intervals (95% CI) are reported. All tests conducted were two-tailed using a predetermined alpha level of 0.05. No adjustments were made for multiple comparisons, as the analyses were exploratory in nature and intended to identify patterns of association rather than test specific hypotheses. Kaplan–Meier survival analysis was used to estimate projected lifetime prevalence of mental disorders based on retrospectively reported age at onset, with final prevalence values derived from the inverse of the final survival probability. Age at onset was recorded to the day and presented with three decimal places, which reduced clustering at whole-year intervals and allowed for more granular estimation of cumulative incidence. Tied survival times were handled using the Efron approximation of the partial likelihood (Grambsch and Therneau, 1994). This approach allows for estimation of cumulative incidence across the life course, accounting for censoring due to age at interview. Rather than revealing the proportion of those exposed to childhood PTEs who have had a mental disorder *by the time of assessment*, projected lifetime prevalence provides an estimate of the proportion who will develop a disorder by the end of their life.

The ‘Survey’ package within R software version 4.3.0 was used and all analyses were conducted in the ABS’s secure DataLab environment. Primary and secondary suppression rules were used for cell with counts of <10 to ensure that no individuals could be identified. All point estimates and SEs were calculated using weights and replicate weights, respectively.

### Ethics approval

This study was deemed exempt from ethical review by the University of Sydney Ethics Office as it meets the criteria of negligible risk research and involves the use of existing collections of non-identifiable data.

## Results

At the time of participating in the survey, those exposed to childhood PTEs were younger compared to those exposed to adult PTEs, but older than those exposed to no PTEs (Table 2). Those exposed to childhood PTEs had significantly higher odds of being female (OR: 1.20, 95% CI: 1.07–1.36), and had experienced a greater number of PTEs overall (mean = 3.45 vs 1.91), compared to those exposed to adult PTEs, *t*(7357) = 39, *p* < 0.001.

**Table 2.** Characteristics of Australian adults exposed to childhood PTEs (CT), adult PTEs only (AT) and no PTEs (NT) (*n* = 15,893).

	Mean scores (SE) or weighted prevalence (%, SE)			*p* (>| *t*|)^a^ or OR (95% CI)	
	CT	AT	NT	CT v AT	CT v NT
Age (M, SD)	44.72 (0.18)	52.79 (0.28)	41.29 (0.26)	<0.01	<0.01
Gender					
% Male	46.92 (0.72)	51.67 (0.97)	49.56 (0.76)	1	1
% Female	53.08 (0.72)	48.33 (0.95)	50.44 (0.76)	1.20 (1.07–1.36)	1.10 (1.00–1.21)
Number of trauma types experienced (M, SD)	3.45 (2.37)	1.91 (1.33)	0	<0.01	NA

Raw cell counts for the ‘non-binary’ gender variable were too low (<10) in some cells and therefore not reported as per ABS reporting restrictions.

a *p* value associated with two-tailed *t*-statistic.

As shown in Table 1, 41.61% (SE = 0.54) of Australians (representing approx. 8,250,948) experienced a childhood PTE, and 70.47% (SE = 0.41) had experienced any PTE in their lifetime (i.e. child and/or adult PTE). Of those who had experienced childhood PTEs, almost one-half (46.25%, SE = 0.85) had experienced multiple childhood PTEs, with a mean number of 1.9 (SD = 1.41) PTEs experienced prior to 18 years. The mean age of first exposure for those who experienced childhood PTEs was 9.55 years (SD = 4.76), with 50% having been exposed by age 10% and 75% by age 14. As reported in Table 1, the more common childhood PTEs were unexpected death of a loved one (27.47%, SE = 0.67), witnessing domestic violence within the home (21.09%, SE = 0.71), experiencing sexual assault (20.98%, SE = 0.67) and witnessing death or serious injury (20.04%, SE = 0.68). The youngest average age for specific types of traumas was 6.88 years (SD = 3.50) for being badly beaten by parent or guardian and 7.13 years (SD = 3.31) for witnessing domestic violence within the home.

### Mental and substance use disorders

As depicted in Table 3, just over half of those exposed to childhood PTEs (53.25%, SE = 0.83) reported experiencing any *DSM*-IV mental or substance use disorder. After controlling for age, gender and number of PTEs experienced, those exposed to childhood PTEs demonstrated significantly higher odds of experiencing any mental or substance use disorder compared to those with no PTEs (adjusted odds ratio [aOR]: 1.51, 95% CI: 1.32–1.74); however, there was no significant difference between those who had experienced childhood PTEs compared to those who experienced adult PTEs (aOR: 1.11, 95% CI: 0.99–1.24).

**Table 3.** Weighted prevalence and associations between lifetime *DSM*-IV mental health and substance use disorders and childhood PTEs (*n* = 15,893), and projected probability of lifetime *DSM*-IV mental health and substance use disorders for those exposed to childhood PTEs.

	Weighted prevalence % (SE)			OR, 95% CI^a^		Weighted projected lifetime probability %, 95% CI
	CT	AT	NT	CT v AT	CT v NT	CT
Panic disorder	6.79 (0.37)	3.09 (0.29)	2.42 (0.32)	**1.31 (1.06–1.63)**	**1.58 (1.16–2.16)**	8.34 (7.17–8.74)
Agoraphobia	5.93 (0.43)	2.60 (0.40)	1.98 (0.45)	1.26 (0.87–1.81)	1.48 (0.84–2.61)	6.73 (5.31–6.63)
Social Phobia	18.52 (0.72)	9.45 (0.61)	9.52 (0.60)	**1.29 (1.09–1.54)**	**1.38 (1.14–1.68)**	19.48 (17.59–19.60)
GAD	14.75 (0.58)	8.07 (0.57)	5.44 (0.52)	**1.20 (1.01–1.44)**	**1.58 (1.17–2.13)**	19.49 (17.37–19.97)
OCD	8.72 (0.40)	3.87 (0.35)	2.44 (0.29)	**1.36 (1.09–1.69)**	**2.32 (1.67–3.23)**	10.55 (8.93–11.16)
PTSD	12.91 (0.56)	5.99 (0.44)	–	1.14 (0.91–1.41)	–	16.98 (14.77–17.77)
Any anxiety disorder	35.40 (0.81)	21.54 (0.86)	15.15 (0.73)	1.12 (0.98–1.27)	**1.53 (1.28–1.81)**	40.69 (38.57–41.79)
Depressive episode	22.15 (0.69)	12.75 (0.76)	8.76 (0.64)	1.11 (0.93–1.32)	**1.34 (1.07–1.67)**	28.13 (26.20–29.23)
Dysthymia	5.78 (0.38)	2.38 (0.25)	1.05 (0.20)	**1.45 (1.09–1.93)**	**2.11 (1.34–3.32)**	8.41 (6.91–8.99)
Bipolar disorder	2.70 (0.2)	0.51 (0.1)	0.82 (0.1)	0.97 (0.30–3.28)	**2.11 (1.07–4.16)**	3.21 (2.42–3.33)
Any affective disorder	22.88 (0.67)	13.22 (0.75)	8.89 (0.64)	1.10 (0.93–.130)	**1.35 (1.09–1.68)**	28.92 (26.93–29.97)
Alcohol use disorder	23.25 (0.60)	16.78 (0.60)	9.10 (0.54)	1.06 (0.93–1.21)	**1.47 (1.24–1.74)**	28.03 (25.77–28.44)
Drug use disorder	6.29 (0.45)	3.19 (0.26)	2.18 (0.31)	1.14 (0.87–1.49)	**1.49 (1.03–2.15)**	7.17 (5.94–7.23)
Any substance use disorder	25.85 (0.69)	17.87 (0.62)	9.90 (0.58)	1.11 (0.98–1.26)	**1.56 (1.33–1.83)**	30.43 (28.06–30.74)
Any *DSM*-IV disorder	53.25 (0.83)	37.43 (1.03)	24.98 (0.85)	1.11 (0.99–1.24)	**1.51 (1.32–1.74)**	59.44 (56.89–59.95)

*DSM*-IV: *Diagnostic and Statistical Manual of Mental Disorders*, Fourth Edition; SE: standard error; OR: odds ratio; CI: confidence interval; CT: childhood PTE; AT: adult-only PTE; NT: no PTEs; GAD: generalised anxiety disorder; OCD: obsessive compulsive disorder; PTSD: post-traumatic stress disorder.

a Adjusting for age, gender and number of PTEs experienced.

The significance of bold values indicates *p* < 0.05.

Australians exposed to childhood PTEs demonstrated significantly increased odds of experiencing panic disorder (aOR: 1.31, 95% CI: 1.06–1.63), social phobia (aOR: 1.29, 95% CI: 1.09–1.54), generalised anxiety disorder (aOR: 1.20, 95% CI: 1.01–1.44), OCD (aOR: 1.36, 95% CI: 1.09–1.69) and dysthymia (aOR: 1.45, 95% CI: 1.09–1.93) compared to those who experienced adult PTEs only and had increased odds of all *DSM*-IV mental disorders (Table 3) compared to those who had not experienced PTEs (ORs: 1.34–2.32), with the exception of agoraphobia. The most common lifetime *DSM*-IV mental disorders experienced by people exposed to childhood PTEs were alcohol or other drug use disorder (25.85%, SE = 0.69), depression (22.15%, SE = 0.69), social phobia (18.52%, SE = 0.72), generalised anxiety disorder (14.75%, SE = 0.58) and PTSD (12.91%, SE = 0.56). Over half of Australians exposed to childhood PTEs were projected to develop a mental disorder in their lifetime (59.44%, 95% CI: 56.89–59.95; Table 3).

### Suicidal thoughts and behaviours

As shown in Table 4, the odds of suicidal thoughts, suicide plans, attempts and self-harm were significantly higher among those exposed to childhood PTEs compared to both the adult PTE and no PTE groups. In fact, people exposed to childhood PTEs had over two times the odds of suicidal attempts compared to those with no PTEs. As shown in Table 4, over a third of Australians exposed to childhood PTEs were projected to experience suicidal thoughts in their lifetime (34.81%, 95% CI: 31.17–35.28) and just over one in 10 were projected to attempt suicide in their lifetime (10.92%, 95% CI: 9.19–10.09).

**Table 4.** Weighted prevalence and associations between lifetime suicidal ideation, suicide plans, suicide attempts and self-harm for those exposed to childhood PTEs (*n* = 15,893), and projected probability of lifetime suicidal ideation, suicide plans, suicide attempts and self-harm for those exposed to child trauma.

	Weighted prevalence % (SE)			OR (95% CI)^a^		Weighted projected lifetime probability %, 95% CI
	CT	AT	NT	CT v AT	CT v NT	Weighted probability % 95% CI
Suicidal thoughts	26.20 (0.77)	11.91 (0.66)	7.96 (0.64)	**1.48 (1.27–1.72)**	**1.57 (1.28–1.92)**	34.81 (31.17–35.28)
Suicide plans	12.53 (0.57)	4.86 (0.45)	2.86 (0.37)	**1.40 (1.11–1.76)**	**1.60 (1.17–2.19)**	18.27 (15.29–17.71)
Suicide attempts	8.77 (0.40)	2.86 (0.31)	1.43 (0.23)	**1.55 (1.20–2.00)**	**2.04 (1.40–2.98)**	10.92 (9.19–10.09)
Any self-harm	14.23 (0.51)	4.80 (0.39)	4.74 (0.39)	**1.39 (1.13–1.72)**	**1.82 (1.42–2.32)**	15.34 (12.86–13.76)

SE: standard error; OR: odds ratio; CI: confidence interval; CT: childhood PTE; AT: adult-only PTE; NT: no PTEs.

a Adjusting for age, gender and number of trauma types experienced.

The significance of bold values indicates *p* < 0.05.

### Physical health

Finally, as illustrated in Table 5, the most common physical health conditions experienced by those exposed to childhood PTEs were arthritis (18.98%, SE = 0.61), followed by asthma (15.21%, SE = 0.56), heart disease (8.40%, SE = 0.44) and diabetes (6.90%, SE = 0.32). There was little evidence of a difference in the odds of all physical health conditions between those exposed to childhood PTEs compared to adult PTEs, after adjusting for age, gender and number of PTEs, with the exception of cancer, for which those exposed to childhood PTEs had lower odds of cancer (aOR: 0.68, 95% CI: 0.54–0.87). Compared to the no PTE group, people exposed to childhood PTEs had significantly higher odds of arthritis, asthma, cancer and kidney disease.

**Table 5.** Weighted prevalence of lifetime physical health conditions for those exposed to child trauma and regression analyses depicting associations with child trauma (*n* = 15,893).

	Weighted prevalence % (SE)			OR (95% CI)^a^	
	CT	AT	NT	CT v AT	CT v NT
Arthritis	18.98 (0.61)	22.90 (0.84)	10.53 (0.49)	1.11 (0.95–1.30)	**1.34 (1.11–1.62)**
Osteoporosis	5.46 (0.34)	7.27 (0.46)	3.25 (0.34)	0.99 (0.77–1.29)	1.00 (0.70–1.43)
Asthma	15.21 (0.56)	12.21(0.65)	8.72 (0.54)	1.04 (0.87–1.25)	**1.24 (1.03–1.50)**
Cancer	5.11 (0.34)	8.36 (0.57)	1.55 (0.18)	**0.68 (0.54–0.87)**	**1.75 (1.24–2.46)**
Diabetes	6.90 (0.32)	9.30 (0.58)	4.36 (0.36)	0.88 (0.72–1.07)	1.01 (0.74–1.38)
Heart disease	8.40 (0.44)	11.35 (0.55)	4.17 (0.35)	0.99 (0.82–1.20)	1.28 (0.96–1.72)
Stroke	1.12 (0.18)	1.63 (0.26)	0.35 (0.11)	0.80 (0.53–1.22)	1.52 (0.64–3.64)
Kidney disease	1.49 (0.16)	1.78 (0.26)	0.51 (0.12)	1.19 (0.83–1.72)	**2.24 (1.19–4.20)**
Bronchitis or emphysema	4.20 (0.31)	3.22 (0.29)	1.66 (0.22)	1.22 (0.94–1.58)	0.97 (0.70–1.34)

SE: standard error; OR: odds ratio; CI: confidence interval; CT: childhood PTE; AT: adult-only PTE; NT: no PTEs.Raw cell counts for ‘dementia’ variable were too low (<10) in some cells and therefore not reported.

a Adjusting for age, gender and number of trauma types experienced.

The significance of bold values indicates *p* < 0.05.

## Discussion

This contemporary, nationally representative study underscores the significant burden of childhood PTEs in Australia, with an estimated 41.6% of Australian adults (approx. 8.25 million people) reporting exposure. Nearly 78% of those exposed to childhood PTEs had experienced multiple types of PTEs, with an average of 3.5 different PTEs in their lifetime and 1.9 PTEs during childhood. Half had experienced PTEs by age 10. The most prevalent childhood PTEs were the unexpected death of a loved one, witnessing domestic violence, sexual assault and witnessing death or serious injury. These experiences were strongly associated with elevated rates of lifetime mental and substance use disorders. In models adjusting for age, gender and the number of PTEs experienced, those exposed to childhood PTEs had higher odds of all mental and substance use disorders, with the exception of agoraphobia, compared to those who had not experienced any PTEs. In addition, those exposed to childhood PTEs had significantly higher odds of disorders such as panic disorder, social phobia, generalised anxiety disorder, OCD and dysthymia, compared to those exposed only to adult PTEs.

The projected lifetime prevalence revealed over half of Australians exposed to childhood PTEs will develop a mental disorder in their lifetime. There was a particularly elevated projected prevalence estimated for the development of anxiety, affective and alcohol use disorders among people exposed to childhood PTEs a finding consistent with other research including longitudinal studies (Bull et al., 2024a; Keyes et al., 2011; Trott et al., 2025). In addition, childhood PTE survivors exhibited a markedly higher risk of suicidal thoughts and behaviours compared to adult and no PTE groups. This is consistent with prior longitudinal research, which finds increased odds of self-harm and suicidal ideation for those exposed to maltreatment in childhood (Kisely et al., 2024). For physical health conditions, those exposed to childhood PTEs had higher odds of arthritis, asthma, cancer and kidney disease, compared to the no PTE group. Unexpectedly, those exposed to childhood PTEs had lower odds of cancer compared to those exposed to adult-only PTEs. This is an interesting finding to be investigated in future research, given the biological embedding of early-life stress that can lead to heightened risk for adverse health outcomes, including cancer (Berens et al., 2017). In this study, it is possible that this finding reflects the younger age of the childhood PTE group compared to adult PTE group, meaning they had not yet lived long enough to reach the ages at which cancers typically have their onset.

These findings confirm strong associations between PTE exposure, particularly in childhood compared to adulthood, and mental and physical health difficulties. Childhood PTEs may have a stronger impact on adult mental and physical health compared to PTEs experienced only in adulthood due to the critical developmental periods during which it occurs (Andersen and Teicher, 2008). Childhood is a time of rapid brain development, with early experiences shaping neural circuits that govern emotional regulation, stress response and cognitive functioning (Lund et al., 2022; Smith and Pollak, 2020). Exposure to PTEs during these formative years can dysregulate the hypothalamic-pituitary-adrenal (HPA) axis, leading to heightened physiological reactivity to stress and long-term alterations in brain structures such as the amygdala, hippocampus and prefrontal cortex (Berens et al., 2017; Smith and Pollak, 2020). Disruptions to the HPA axis have flow on effects of a range of interconnected biological systems, including the immune and metabolic systems, increasing the risk of chronic disease and mood disorders (Sheng et al., 2021). While both childhood and adult PTEs may invoke self-medication with substances or highly palatable, calorie-dense food (Hemmingsson, 2014), these lifestyle risk behaviours occurring early in the life course may compound over time, resulting in greater odds of harmful outcomes associated with earlier exposure to PTEs. Childhood PTEs may also disrupt the formation of secure attachments and hinder the development of protective social and emotional skills, further exacerbating its impact into adulthood (Baer and Martinez, 2006; Groh et al., 2014).

These findings represent the most up-to-date, nationally representative prevalence estimates of PTEs in the Australian population. There was some overlap in the trauma types measured here and the ACMS, with childhood physical abuse and domestic violence collected in both studies (Haslam et al., 2023). For both exposures, our study found a lower prevalence: 22% exposure to domestic violence compared to 40% in the ACMS, and 12.3% physical abuse compared to 32% in ACMS (Mathews et al., 2023). This may be due to this study assessing each PTE with a single item. For domestic violence, this was ‘ever witnessed any serious physical fights at home when a child’. In contrast, the ACMS included items for exposure to physical violence between parents, serious threats made against a parent by a partner, property damage during parent arguments as well as intimidation or control verbally, sexually, financially or by isolating them from social support (Haslam et al., 2023). These more comprehensive items in the ACMS capture a broader spectrum of behaviours involved in domestic violence compared to the single item in the NSMHW. For physical abuse, the item used in this study was ‘ever badly beaten as a child by parents/guardians’. ACMS collected this information regarding any adult, i.e., whether an adult ever physically hurt them, potentially explaining the greater prevalence than what was found in this study (Haslam et al., 2023). In addition, the response rate for the ACMS was very low (4%), compared to the response rate of 52% of the NSMHW (ABS, 2020-2022; Haslam et al., 2023). If the decision to respond to the ACMS was related to the experience of maltreatment, this could have skewed the prevalence of exposure. Given the relative strengths of the ACMS item set, particularly its comprehensive coverage of maltreatment types, there may be value in considering the integration of selected items into future iterations of the NSMHW. This could enhance the depth and comparability of maltreatment data across national surveys, supporting more consistent prevalence monitoring and facilitate linkage with other datasets where longitudinal follow-up is possible.

The findings of this study reinforce that childhood PTEs are a national public health issue with far-reaching implications. The high prevalence of childhood PTEs, coupled with significant associations with mental disorders, suicidality and physical health, underscores the critical need for systemic responses that prioritise early identification, prevention and intervention. Trauma-informed approaches must be at the forefront of efforts to address these complex and interconnected outcomes. This includes embedding trauma recognition and response into the policies and practices of health care, education, child welfare, justice systems and other frontline services to create environments that support recovery and resilience (Ko et al., 2008).

## Limitations

This study has several limitations. The reliance on retrospective self-report for PTEs may introduce bias, as individuals’ experience of mental illness may influence their subjective appraisal of past experiences, potentially over-inflating associations between childhood PTEs and mental disorders. Indeed, retrospective self-report of childhood PTEs typically shows stronger associations with mental health compared to prospectively assessed childhood PTEs (Danese and Widom, 2020; Reuben et al., 2016). This bias may be tempered in this study, as we measured associations with lifetime mental disorders rather than current conditions, and associations between childhood PTEs and physical health are less dependent on whether PTEs measured prospectively or retrospectively (Reuben et al., 2016). The cross-sectional design of this study limits the ability to infer causal relationships between childhood PTEs and the reported lifetime diagnoses of mental and substance use disorders. While the findings demonstrate strong associations, longitudinal data would provide a more robust understanding of the temporal relationships between PTEs and subsequent outcomes. The use of Kaplan–Meier survival analysis with retrospectively reported age at onset introduces several limitations. Although age at onset was recorded to the day and presented with three decimal places, reducing clustering and improving granularity, recall bias remains a concern, as participants may inaccurately recall the timing of disorder onset. In addition, survival bias may occur if individuals with earlier onset or more severe conditions are underrepresented due to mortality-related right-censoring. Cohort and period effects may also influence survival patterns, as risk of disorder onset can vary by historical context and birth cohort, not just age. Importantly, these limitations are likely to result in conservative estimates of lifetime prevalence. Prospective studies consistently find earlier age of onset and higher cumulative prevalence than retrospective surveys (Moffitt et al., 2010; Takayanagi et al., 2014), suggesting that our projections may understate rather than overstate the true burden of mental disorders across the lifecourse. In addition, while we adjusted for age, gender and the number of PTEs, the NSMHW only collected information about the participant’s socioeconomic status (SES) at the time of the survey and not during childhood. Thus, we did not control for SES, which likely influences the relationships reported through cumulative disadvantage and harm (Evans and Cassells, 2014; Seabrook and Avison, 2012), nor could we control for other relevant risk factors for childhood PTEs that were not available in NSMHW data, such as maternal social isolation in the postpartum period, low maternal education and young pregnancies (Bull et al., 2024b). Furthermore, we did not adjust for multiple comparisons, which increase the risk of type I error. As such, findings should be interpreted with caution and considered hypothesis-generating rather than confirmatory. Another limitation of this study is the representativeness of the sample. The NSMHW is a household-based sample and excludes individuals experiencing homelessness, incarceration or institutional care. These groups are likely to have higher rates of PTEs, mental disorders and physical health concerns, which could affect the relationships explored in this study and the generalisability of our findings to these vulnerable populations. In addition, the NSMHW’s response rate of 52% raises concerns about potential non-response bias. The characteristics and experiences of the nearly half of the population who did not participate may differ significantly from those included in the analysis, potentially limiting the broader applicability of the results. The lack of representation of very remote areas and discrete Aboriginal and Torres Strait Islander communities is of particular concern, limiting its generalisability to First Nations populations. Future research examining childhood PTEs among Aboriginal and Torres Strait Islander peoples is essential and must be conducted in genuine partnership with communities, using culturally safe and appropriate methodologies. Furthermore, the study did not assess personality disorders, which are often associated with PTEs, nor did it use the updated *DSM*-5 diagnostic criteria, which may affect the generalisability of the findings to current clinical practice and frameworks.

## Conclusion

This study highlights the pervasive and enduring impact of childhood PTEs on mental and physical health, as well as suicidality, underscoring the urgent need for coordinated, trauma-informed approaches to mental and physical healthcare.
